# Increasing complexity in vascular microphysiological systems: considerations and advances for disease modeling

**DOI:** 10.3389/fbioe.2026.1927687

**Published:** 2026-09-28

**Authors:** Katerina Katsouleas, Jennifer H. Hammel, Sharon Gerecht

**Affiliations:** Department of Biomedical Engineering, Duke University, Durham, NC, United States

**Keywords:** biomaterials, hemodynamic flow, iPSC, organ-on-chip, vasculature hierarchy

## Abstract

Microphysiological systems (MPS) and organ-on-chip (OoC) platforms are miniaturized *in vitro* models of human tissues that have transformed approaches to disease modeling and drug screening. Compared to traditional cell culture systems, MPS/OoC enable precise control over parameters such as tissue architecture, cell-cell interactions, and biophysical and biochemical cues, allowing recapitulation of native tissue microenvironments and mechanistic studies of disease progression. In the vascular field, MPS have been developed across multiple levels of the vascular hierarchy, including microvascular capillary networks, arteriole- and venule-scale systems, and larger artery and tissue-engineered blood vessel models. These platforms support the investigation of diverse vascular pathologies, ranging from macrovascular disease, such as atherosclerosis, to microvascular dysfunction and barrier failure. In this review, we summarize key engineering and biological considerations for, and pivotal advancements in, vascular MPS development with added complexities such as informed cell sourcing strategies, incorporation of mural and immune populations, the selection and use of dynamic biomaterials, sustained hemodynamic, pulsatile flow, and architectures tailored to specific vascular scales. We conclude by outlining future considerations, including integrating models across the vascular hierarchy, expanding organ-specific vascular platforms, and leveraging single-cell transcriptomics to improve mechanistic analysis and support rational model design.

## Introduction

The human vascular system is a hierarchically organized and dynamic network responsible for the distribution of oxygen, nutrients, hormones, and immune mediators throughout the body. This system spans multiple spatial scales, from large conduit vessels (arteries and veins), through intermediate resistance vessels (arterioles and venules), to the microvasculature, where dense capillary networks–often consisting of single-cell-thick endothelial lumens–mediate direct exchange with surrounding tissues. Despite forming a continuous circulatory network, vessels at each hierarchical level are defined by distinct cellular compositions, extracellular matrix (ECM) architectures, mechanical properties, and hemodynamic environments (Margolis et al., 2023). The field of vascular engineering aims to recapitulate these diverse hierarchies to better understand vascular function and disease.

Large arteries and veins exhibit a multilayered organization consisting of a tunica intima, tunica media, and tunica adventitia. The innermost layer, the tunica intima, is composed of an endothelial cell (EC) monolayer. Moving outward, there is increased complexity. The tunica media has circumferentially aligned smooth muscle cells (SMCs) and elastic ECM (though arteries have far greater SMC and ECM coverage than veins), and the tunica adventitia contains fibroblasts, connective tissue, and resident immune cells (Fleischer et al., 2020; Zhang L. et al., 2025). Mesovasculature, like small veins, arterioles, and venules, also retains endothelial and mural cell layers but has reduced wall thickness, fewer SMC layers, and reduced or no elastic lamellae, reflecting their role in flow regulation rather than bulk transport (Mallat et al., 2016). At the smallest vascular scale, capillaries possess a thin basement membrane and lack SMC coverage, relying on pericytes for structural support, barrier regulation, and tissue coupling (Marchand et al., 2019) (Figure 1A). Distinct from the blood vasculature, lymphatic vasculature provides waste drainage and fluid balance, transporting lymph. Initial lymphatic vessels are capillary-scale and lack basement membranes, filtering interstitial fluid that then travels to larger collecting lymphatic vessels, which often have smooth muscle cells (Suarez et al., 2023). Each vascular system serves distinct roles, but often shares key structural features.

**FIGURE 1:**
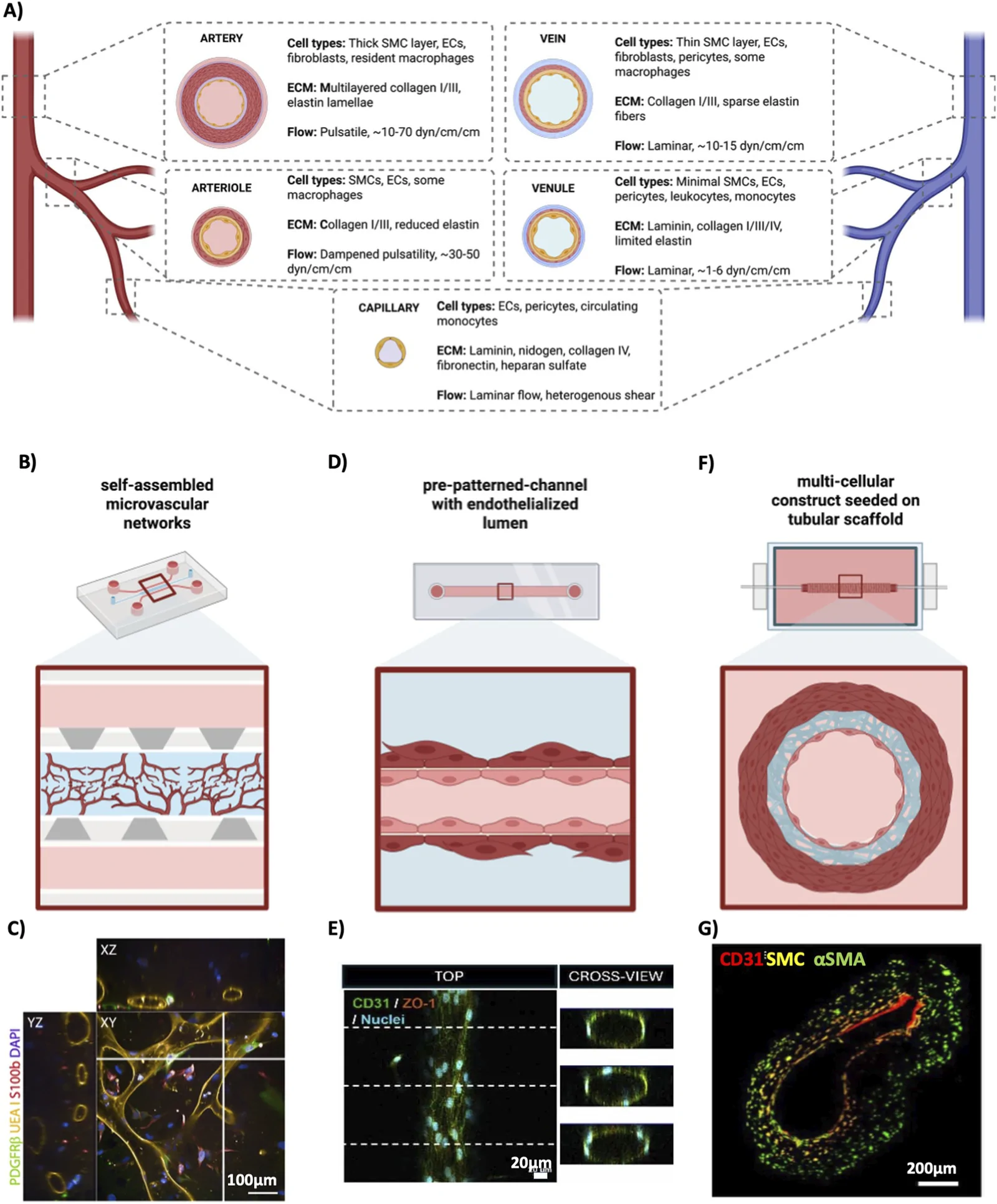
Vascular MPS aim to replicate the human vascular complexity across hierarchical scales. **(A)** Schematic of the vascular hierarchy from macro-vessels (arteries and veins), to meso-vessels (arterioles and venules), to micro-vessels (capillaries) with relevant characteristics to inform model design. **(B–G)** Illustration of various microfluidic culture methods employed across vascular MPS at different scales and representative immunofluorescence images of each. Example schematic of an MPS with a self-assembled microvascular network **(B)** and representative fluorescent image from an inner-blood-retinal-barrier on a chip **(C)** scale bar is 100 microns. Panel C was reproduced from Maurissen et al. (Maurissen et al., 2024) under the creative commons license found here: http://creativecommons.org/licenses/by/4.0/. **(D)** Example schematic of an MPS with a pre-patterned endothelialized channel and **(E)** surrounding mural cells and a representative fluorescent image from a 3D printed pre-patterned capillary vessel on a chip. Scale bar is 20 microns. Panel E was reproduced from Salvadori et al. (Salvadori et al., 2025) under the creative commons license found here: http://creativecommons.org/licenses/by/4.0/. **(F)** Example schematic of an MPS cannulated with a self-supporting, multilayered vessel on a tubular scaffold and **(G)** a representative fluorescent image from an artery-on-chip containing both ECs (red) and SMCs (yellow/green). Scale bar is 200 microns. Panel G was reproduced from Zhang et al. (Zhang et al., 2020) under the creative commons license found here: http://creativecommons.org/licenses/by/4.0/.

Across the vascular hierarchy, endothelial and mural cell phenotypes vary markedly, as do patterns of shear stress, pulsatility, and circumferential strain, giving rise to vessel-specific functions and adaptive responses (Dessalles et al., 2021; Liu et al., 2025). As a result, vascular disease manifests in a scale-dependent manner: atherosclerosis, aneurysm formation, hypertension, and venous thrombosis disease predominantly affect macro-vessels, and their manifestations include vessel weakness, plaque accumulation, intimal hyperplasia, and calcification. Contrastingly, diseases like diabetic retinopathy, coronary microvascular disease, vascular dementia, and chronic kidney disease manifest in the microvasculature and are characterized by barrier breakdown, aberrant angiogenesis or vessel regression, impaired perfusion, and altered immune trafficking (Liu et al., 2025). Traditional *in vitro* models however, have generally studied vascular cell types in isolation (i.e., ECs only or SMCs only) and in static, 2D environments. These simplified systems do not reproduce the mechanical forces (shear stress, strain), multicellular crosstalk, or 3D geometry that distinguish one part of the vascular tree from another, and thus fail to capture the scale-dependent heterogeneity in vascular disease. This mismatch between model simplicity and disease complexity limits our mechanistic understanding of vascular pathophysiology and motivates the need for models that integrate multicellular interactions and tissue-specific microenvironments and geometries.

To increase physiological relevance *in vitro*, vascular microphysiological systems (MPS), also referred to as organ-on-chip (OoC) platforms, have been developed as miniaturized culture models that incorporate tissue-like architectures, multiple cell types, 3-dimensional ECM environments, and controlled fluid flow (Ingber, 2022). In particular, the inclusion of perfusion distinguishes vascular MPS from conventional culture systems, enabling regulation of shear stress, mass transport, and mechanotransduction. Collectively, these systems have enabled disease modeling, mechanistic interrogation of vascular biology, drug screening, and patient-specific studies, while offering a complementary alternative to animal models that reduces cost and mitigates interspecies differences (Zhang et al., 2018; Haddrick and Simpson, 2019; Pollet and Toonder, 2020). A detailed history of the development of these platforms, as well as their applications in disease modeling to date, can be found in recent thorough reviews (Liu et al., 2025; Zhang et al., 2020; Shelton, 2024; Lim et al., 2024).

Contemporary vascular MPS span multiple vascular scales, with varied approaches for each scale (Figure 1A). Microvascular models are commonly embedded within soft ECM-like hydrogels (e.g., collagen or fibrin), where patterned channels or self-assembled endothelial networks form perfusable capillary-like structures (Figures 1B,C; Table 1) (Maurissen et al., 2024). Arteriole- and venule-scale models often utilize open channels patterned within matrices, seeding SMCs or pericytes and ECs sequentially to establish an endothelialized, perfusable lumen (Figures 1D,E) (Salvadori et al., 2025). In contrast, arterial MPS, which must tolerate substantially greater pressures and cyclic strain, typically rely on more mechanically robust, tubular scaffolds–often fabricated from synthetic materials–rather than containment within a soft matrix on a sealed device or chip (Figures 1F,G; Table 1) (Zhang et al., 2020).

**TABLE 1:** Summary of MPS design properties across vascular scales**.** Native ECM components, mechanics, and the materials used to model vessels of varying scale within the vascular hierarchy. Materials selection is summarized from the referenced studies.

Vascular scale	Native ECM composition	Effective stiffness range	Dominant mechanical cues	Commonly used materials in MPS	References
Microvasculature (capillaries, post-capillary venules)	Basement membrane enriched in laminin (411/511), collagen IV, nidogen, perlecan; minimal fibrillar collagen; extensive pericyte-EC interactions (Marchand et al., 2019; Wang et al., 2023)	∼0.1–5 kPa (Bjorgvinsdottir et al., 2024)	Moderate shear stress (∼1–100 dynes/cm^2^, highly heterogeneous), minimal circumferential strain, matrix viscoelasticity, permeability gradients, hypoxia (Martier et al., 2024)	Collagen I/IV	(Lin et al., 2026; Buchanan et al., 2013; Brandenberg and Lutolf, 2016; Sano et al., 2022; Bae et al., 2022)
				Fibrin	(Kim et al., 2021; Chen S et al., 2023; Chen et al., 2009; Whisler et al., 2014; Li et al., 2022; Isosaari et al., 2023; Bains et al., 2025; Vila Cuenca et al., 2021; Berry et al., 2021)
				Matrigel	(Huang and Lei, 2022; Carvalho et al., 2019)
				Hyaluronic acid	(Natividad-Diaz et al., 2019; Schnellmann et al., 2022; Song et al., 2025; Wei et al., 2023; Wei et al., 2020; Blatchley et al., 2019; Hanjaya-Putra et al., 2011)
				Composite materials	(Liu et al., 2021)
Mesovasculature (veins, small arteries, arterioles, venules)	Mixed ECM with collagen I/III, elastin, proteoglycans; discontinuous internal elastic lamina; SMC coverage (Mallat et al., 2016; Wang et al., 2023)	∼5–50 kPa (Bjorgvinsdottir et al., 2024)	Moderate shear (20–50 dynes/cm^2^), vasoactive strain, cyclic stretch (5%–10%), ECM fiber alignment (Martier et al., 2024)	Collagen I	(Zhang et al., 2020; Zheng et al., 2012; Brandenberg and Lutolf, 2016; Zhao et al., 2012; Akther et al., 2025; Su et al., 2021; Paloschi et al., 2024)
				Fibrin (electrospun or bulk hydrogel)	(Yarbrough et al., 2026; Chen S et al., 2023; Zhang et al., 2016)
				Natural materials (elastin, gelMA, chitosan, *etc.*- excluding collagen and fibrin	(Colosi et al., 2016; Wu et al., 2021; Gu et al., 2022)
				Synthetic materials	(Rajeeva Pandian et al., 2020; Baksamawi et al., 2023; Weener et al., 2024; Millik et al., 2019; Yasotharan et al., 2015)
Macrovasculature (arteries)	Multilayered ECM with elastin lamellae, dense collagen I/III bundles, proteoglycans; pronounced anisotropy in the case of arteries (Zhang et al., 2025a; Wang et al., 2023)	∼50 kPa-1 MPa (non-linear) (AMRPN, 2013; Bjorgvinsdottir et al., 2024)	High pulsatile shear (10–70 dynes/cm^2^), cyclic circumferential strain (5%–15%), pressure-driven non-linear elasticity (Martier et al., 2024)	Electrospun fibrous scaffolds	(Lovett et al., 2010; Joy et al., 2018; Li et al., 2020; Fahad et al., 2024)
				Natural hydrogels (often chemically reinforced)	(Maringanti et al., 2024; Mastoor et al., 2024; Helms et al., 2021; Li et al., 2017; Huynh and Tranquillo, 2010; Ryan et al., 2020)
				Decellularized scaffolds	(Cho et al., 2005; Hsia et al., 2019)
				Synthetic materials	(Sharifpoor et al., 2011; Rayatpisheh et al., 2014; Xing et al., 2017; Pezzoli et al., 2017)

The state-of-the-art vascular MPS typically involves 3-dimensional cultures of microvascular networks or macroscale materials to mimic hollow, large-scale vessels. These models have greater physiological relevance than traditional 2D cultures and can serve as high-throughput platforms for addressing biological questions or screening therapeutics. However, despite the advantages vascular MPS offer, improvements are still necessary to increase relevance, such as incorporating additional vascular cells beyond ECs and SMCs, increasing tissue specificity, maintaining continuous perfusion, and enhancing control over MPS biomechanics. In this review, we focus on emerging strategies to increase biological and physical complexity in vascular MPS. Specifically, we examine advances in cell selection and sourcing, informed and dynamic biomaterial selection, implementation of physiologically relevant and sustained flow regimes, and how these parameters should be tuned according to the biological question being asked or the disease being modeled. We end by discussing integration across vascular scales and multi-organ platforms, highlighting representative examples and outlining future directions to enhance the throughput and utility of vascular MPS in disease modeling and translational research.

### Cell selection

A clear strategy to increase complexity in vascular MPS lies in cell selection. Many vascular MPS platforms rely on readily available, well-characterized cell types–such as human umbilical vein endothelial cells (HUVECs), endothelial colony-forming cells, human aortic smooth muscle cells, and human dermal fibroblasts–to facilitate platform development and benchmarking (Shakeri et al., 2023). These sources are especially useful for the development of models whose pathological phenotypes may be induced via exogenous stressors that recapitulate disease-relevant cues (e.g., hypoxia, elevated shear or pressure, lipid exposure, oxidative stress) or, for monogenic disorders (e.g., LMNA-associated progeroid syndromes) through targeted genetic perturbation (Ciciliano et al., 2015; Gonzalo et al., 2017; Denecke et al., 2022; Ntekoumes et al., 2025; Maringanti et al., 2024). However, as vascular MPS are increasingly applied to modeling tissue-specific physiology and complex disease states, limitations, including batch-to-batch variability, limited vascular bed specificity, and genetic heterogeneity between constituent cell types, increasingly offset the advantages of their accessibility.

For diseases without a clearly defined genetic driver, patient-derived primary cells may offer greater physiological fidelity (Inamoto et al., 2010; Al-Hilal et al., 2020), but are constrained by limited availability and low yield, particularly for endothelial populations. To overcome these barriers, patient-derived induced pluripotent stem cells (iPSCs) are a scalable alternative, enabling patient-specific vascular cell derivation for advanced MPS applications (Browne et al., 2021; Zhang et al., 2021; Naderi-Meshkin et al., 2024; Marder et al., 2024). Protocols for differentiating standard endothelial cells (iECs), lymphatic endothelial cells (iLECs) and smooth muscle cells (iSMCs) from iPSCs are well established (Patsch et al., 2014; Kurokawa et al., 2017; Natividad-Diaz et al., 2019; Shen et al., 2021; Meijer et al., 2023; Lee et al., 2015; Owsiany et al., 2024), however, increasing emphasis is being placed on the need to characterize and incorporate tissue-specific iECs and iSMCs in *in vitro* modeling. Notably, Rosa et al. showed that iPSC-derived arterial and venous ECs exhibit phenotypic and functional differences in permeability and leukocyte adhesion (Rosa et al., 2019). This is further reinforced by recent single-cell RNA-seq data from the Human Vascular Atlas, which highlights transcriptomic differences not only between arterial and venous ECs, but between subpopulations of arterial ECs (Barnett et al., 2024). Beyond arteriovenous identity, ECs display pronounced heterogeneity across specialized microvascular beds, including the blood-brain barrier, retinal vasculature, and lymphatic endothelium, reflected in differences in junctional organization, barrier permeability, transporter expression, and inflammatory responsiveness (Barnett et al., 2024; Marcu et al., 2018; Cleuren et al., 2019). For example, it has been shown that human brain vascular ECs are resistant to shear-induced alignment and elongation when compared to arterial or venous ECs (Reinitz et al., 2015). iEC differentiation protocols increasingly leverage these transcriptional and signaling cues through modulation of VEGF, Notch, Wnt, and shear-dependent pathways to generate hierarchy- and organ-specific iEC populations with improved fidelity for disease modeling (Owsiany et al., 2024; Oh et al., 2021; Pan et al., 2025; Stern et al., 2025).

For mural cells, iSMC phenotype is strongly influenced by differentiation strategy and culture conditions. In the body, SMCs within the vascular system originate from varying embryonic lineages depending on tissue type; SMCs in the cerebral vasculature and the carotid arteries originate from neural crest progenitors, whereas SMCs in the coronary arteries differentiate from the epicardium (Kwartler and Pinelo, 2024). *In vitro* studies show that these populations of SMCs exhibit differential phenotypes and responses to external stimuli like transforming growth factor β (TGFβ) (Thieszen et al., 1996; Gadson et al., 1997; Pfaltzgra et al., 2014), suggesting that iSMC phenotype may be highly dependent on the method and lineage of differentiation. SMCs are also known for their high plasticity in the body, posing challenges to recapitulating native heterogeneity in iSMCs (Chen et al., 2023; Ma et al., 2025). For example, many iSMC differentiation protocols yield proliferative, synthetic (matrix-producing) or immature cells rather than mature, contractile, quiescent smooth muscle cells. This introduces potential misclassification with myofibroblast-like populations, particularly when α-smooth muscle actin (αSMA) is used as the sole identity marker, and raises concerns about the ability to capture healthy phenotypes (Kwartler and Pinelo, 2024; Gan et al., 2007). Improved iSMC differentiation approaches targeting the contractile phenotype leverage metabolic conditioning (e.g., glucose deprivation), prolonged maturation stages, treatment with TGF-β, and, in some select protocols, the use of MEK or ALK5 inhibitors (Kumar et al., 2017; Gao et al., 2021; Shen et al., 2022; Liu L. et al., 2023). An optimal vascular MPS should incorporate the appropriate iSMC lineage for its intended application, support the maintenance of a contractile phenotype under homeostatic conditions, and permit controlled phenotypic switching in response to disease-relevant stimuli.

While ECs and SMCs are key cell types used in vascular MPS, pericytes and fibroblasts are also critical for vascular structure and function (Figure 2A). Pericytes, typically found in direct contact with ECs, play critical roles in microvascular stabilization, regulation of endothelial barrier function, and capillary perfusion. In diseases such as diabetic retinopathy and neurovascular degeneration, pericyte detachment or loss, rather than primary EC dysfunction, is a hallmark (Antonetti et al., 2021; Li and Fan, 2023). *In vitro* studies by Dibble et al. and Yamamoto et al. demonstrated that pericyte addition alters the structure and maturity of HUVEC networks (Figure 2B) (Yamamoto et al., 2019; Dibble et al., 2023). Additionally, in a recent study, we showed that pericyte-EC interactions can promote greater barrier function in ECs *in vitro* (Lin et al., 2025). Fibroblasts, a major mediator of ECM across the body, are known contributors to vascular health and dysfunction, namely, through vessel stiffening and fibrotic remodeling (Harrison et al., 2021; Schickling and Miller, 2021). Studies using fibroblast-EC or fibroblast-SMC co-cultures have shown that fibroblast paracrine signaling can drive or modulate vascular response to oxidative stress, nitric oxide signaling, intimal thickening, hypertension, and reduced vascular compliance, motivating their incorporation into vascular MPS design (Shakeri et al., 2023; Pauty et al., 2021; Streutker et al., 2024; Luu et al., 2023; Crnkovic et al., 2025). Tissue-specific and disease-specific fibroblasts have also been incorporated into lymphatic MPS to better represent the tissue microenvironment (Hammel et al., 2025; Lugo-Cintron et al., 2021). Although fibroblasts have long been incorporated into microvascular models, they are increasingly recognized as critical components of macrovascular MPS as well (Zhang et al., 2020; Mackay et al., 2022). Notably, recent protocols have been developed to produce iPSC-derived, *vascular-specific* pericytes and fibroblasts, thereby increasing physiological relevance (King et al., 2024; Park et al., 2023).

**FIGURE 2:**
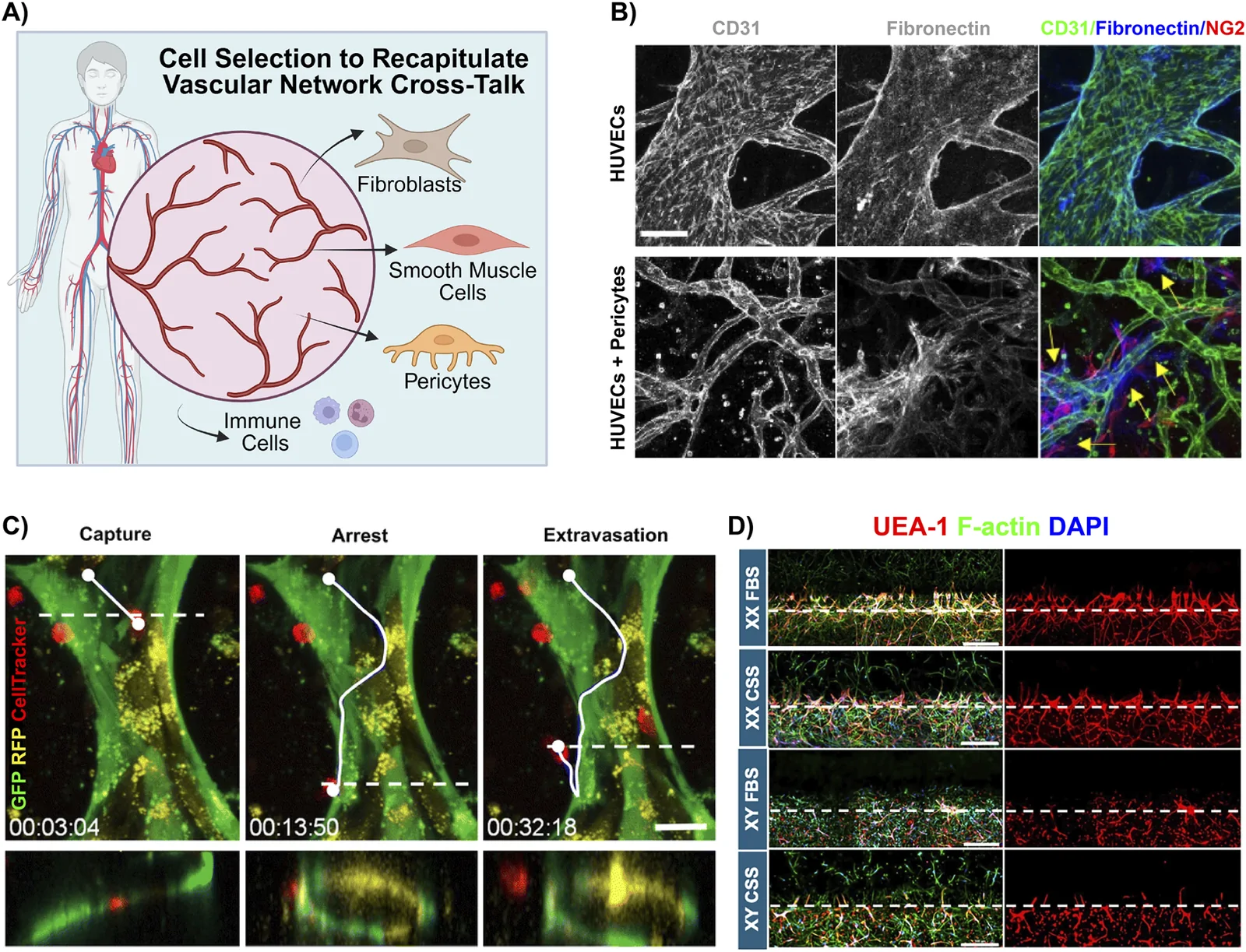
Cell selection is an essential component of capturing vascular function in MPS. **(A)** Vascular networks experience crosstalk with a range of cell types in the body. **(B)** Co-culture between human umbilical vein endothelial cells (HUVECs) and pericytes alters network formation. Scale bar is 100 microns. Panel B was reproduced from Dibble et al. (Dibble et al., 2023) under the Creative Commons license, found here: http://creativecommons.org/licenses/by/4.0/. **(C)** Vascular MPS systems can capture leukocyte adhesion to, migration on, and extravasation through vascular networks. Panel C was reproduced from Hirth et al. (Hirth et al., 2024) under the Creative Commons license, found here: https://creativecommons.org/licenses/by-nc/3.0/. **(D)** Differences in vascular networking among female (XX) and male (XY) endothelial cells can be observed using hormone rich fetal bovine serum (FBS) or hormone-depleted charcoal-stripped serum (CSS). Scale bar is 500 microns. Panel D was reproduced from Martier et al. (Martier et al., 2025) under the Creative Commons license, found here: http://creativecommons.org/licenses/by/4.0/.

Immune cells are integral to the vascular niche, with many vascular pathologies manifesting in altered leukocyte adhesion, transmigration, and inflammatory signaling, particularly in arterial plaque formation and microvascular inflammation and leakage. In an *in vitro* system, the timing of co-culture, activation state, and polarization of immune cells can all impact vascular function. Macrophages can enhance or impede vascular networking *in vitro,* depending on pro-regenerative or pro-inflammatory polarization (Moore et al., 2017; Moore et al., 2018; Graney et al., 2020). Peripheral blood mononuclear cells can enhance vascular permeability when combined with inflammatory signals (Gogesch et al., 2025; Ehlers et al., 2023; Rengarajan et al., 2024). However, the sourcing and maintenance of immune cells remains a logistical challenge (Hammel et al., 2022). While immortalized immune cells and cancer-derived immune cells are frequently used *in vitro*, primary immune cells are necessary to fully recapitulate vascular phenotypes. Primary immune cells are usually isolated fresh from blood using traditional density gradient separation techniques, which then require further purification of the immune cells of interest (McCloskey et al., 2022). Additionally, animal-derived serum, typically used in culture media, can impact immune cell phenotype, leading to widespread use of serum-free media formulations or using heat-inactivated serum (Cochrane et al., 2024; Huang et al., 2025). Finally, primary immune cells have limited longevity in culture, typically lasting a few weeks (Hammel et al., 2022). The maximum culture time for primary immune cells varies widely based on cell type and the addition of stimulatory factors. Cryopreservation of primary immune cells can increase flexibility but can impair post-thaw function without careful consideration (Qi et al., 2024). One solution is the use of tumor-derived immune cells, such as Jurkat T cells and THP-1 monocytes from leukemia patients, that retain aspects of normal immune function (Chanput et al., 2014; Carrasco-Padilla et al., 2023). However, immortalized immune cells can show distinct differences in cytoskeletal dynamics, cell-cell signaling, phagocytosis, and heterogeneity (Bartelt et al., 2009; Colin-York et al., 2019; Ding et al., 2025). Thus, immune cell selection requires careful consideration. Nonetheless, immune cells of varying sources have begun to be incorporated into vascular MPS. Monocytes have been incorporated at the macro-vessel scale through the development of atherosclerosis-on-a-chip models by Caroline Cheng’s and George Truskey’s groups, investigating the mechanisms of monocyte adhesion to and extravasation through the vascular wall during early atherosclerotic lesion formation (Zhang et al., 2020; Maringanti et al., 2024). Leukocytes are more frequently incorporated into lymphatic MPS (Serrano et al., 2024; Jo et al., 2025; Morrison et al., 2025) or vascularized tumor MPS (Zhao et al., 2024; Skubal et al., 2025), but studies have shown recapitulation of leukocyte migration in vascular MPS (Lamberti et al., 2014; Hirth et al., 2024). Hirth et al. demonstrated that leukocytes could traffic through a self-assembled microvasculature-on-chip system, showing the point of leukocyte adhesion, migration along the vessel, and extravasation (Figure 2C) (Hirth et al., 2024). Boosting the future potential of immune incorporation, robust protocols for producing iPSC-derived macrophages have been developed (Spiller et al., 2016; Takata et al., 2017; Ackermann et al., 2022), and iPSC-derived lymphocytes have been established (Ando and Nakauchi, 2017; Nianias and Themeli, 2019; Furukawa et al., 2023), but their incorporation into engineered models is still nascent. These protocols could enable the generation of fully isogenic, immunocompetent vascular models. Before this future can be realized, iPSC-derived immune cells must demonstrate the correct phenotype, sufficient function, and genetic stability (Nianias and Themeli, 2019; Fang et al., 2024).

Apart from cell identity and differentiation state, other essential considerations in cell sourcing include biological sex, age, and ancestral background. Sexual dimorphism has been documented across multiple vascular-relevant cell types, including ECs and SMCs and macrophages, with sex-specific differences in inflammatory signaling, contractility, and stress responses potentially underlying observed disparities in cardiovascular disease (CVD) (McClain et al., 2024; James and Allen, 2021). Martier et al. recently demonstrated that sprouting angiogenesis is impacted by hormone-rich (fetal bovine serum, FBS) and hormone-depleted (charcoal stripped serum, CSS) serums in a sex-specific manner using sex-matched male and female EC-fibroblast 2D and 3D co-cultures (Figure 2D) (Martier et al., 2025). Accordingly, sex-matched or sex-stratified vascular MPS are increasingly recognized as essential for accurately modeling disease phenotypes and therapeutic responses.

Similarly, cellular age profoundly influences vascular function (Propson et al., 2021); microvascular models assembled from aged versus young endothelial cells exhibit significant differences in barrier integrity, reflecting age-associated alterations in junctional organization and mechanotransduction (Hetzer and Bersini, 2022). Recent methodological frameworks have further outlined strategies for modeling vascular aging *in vitro*, including replicative senescence, genetic perturbations (i.e., TERF2 or SIRT6 knock outs), and chronic exposure to oxidative or inflammatory stressors (Wang et al., 2022; Linden et al., 2023; Neherin et al., 2026). In parallel, ancestral background remains a largely underreported variable in regenerative engineering and MPS development, despite mounting evidence that genetic ancestry influences vascular biology and disease susceptibility, underscoring the need for improved reporting and intentional inclusion of diverse donor populations (Ryan et al., 2021). These factors are especially pertinent to consider when designing vascular MPS as age, sex, and genetic predisposition are significant risk factors for a majority of vasculopathies and are necessary components to comprehensive disease modeling.

### Biomaterial selection, tissue mechanics, and vascular geometries

Biomaterial selection is central to MPS design, requiring the concurrent replication of tissue-specific microenvironmental composition and mechanical properties that both approximate native tissues and tolerate physiologically relevant biophysical loading. This is especially true for vascular MPS, as the composition, shear, and loading of the vascular microenvironment vary widely across vessel scale and tissue type. In the microvasculature, ECs reside on a thin basement membrane rich in laminin (particularly laminin-411/511), collagen IV, fibronectin, nidogen, and heparan sulfate proteoglycans, with minimal or absent smooth muscle coverage and extensive pericyte interactions. These capillaries experience a range of shear stresses and stiffnesses across various tissue interfaces, and minimal circumferential strain, allowing for robust barrier function, immune trafficking, and metabolic exchange (Marchand et al., 2019; Wang et al., 2023). In contrast, meso-scale vessels such as small arteries, veins, arterioles, and venules exhibit a more complex ECM architecture, including fibrillar collagens (types I and III), elastin, and proteoglycans, and are exposed to greater pulsatility and vasoactive strain than capillaries (Mallat et al., 2016). Macrovascular tissues, specifically large arteries, are embedded within multilayered ECM environments dominated by elastin-rich lamellae, collagen bundles, and mechanically anisotropic architectures that support high pressures, non-linear elasticity, and high cyclic tensile strain (Zhang L. et al., 2025; Dalbosco et al., 2024; AMRPN, 2013). These hierarchical differences define not only the biochemical cues presented to vascular cells but also the mechanical boundary conditions that regulate cell phenotype, mechanotransduction, and disease susceptibility (Marchand et al., 2019; Yanagisawa and Yokoyama, 2021).

Accordingly, biomaterial selection and mechanical design strategies in vascular MPS have diverged based on the level of the vascular hierarchy being modeled. Micro- and meso-vascular MPS commonly employ soft hydrogels (elastic moduli typically <5 kPa) composed of collagen I, fibrin, gelatin methacryloyl (GelMA), hyaluronic acid (HA), or synthetic and semi-synthetic matrices, to support vasculogenesis, lumen formation, and immune cell transmigration (Table 1) (Lim et al., 2024; Chang et al., 2026). Hydrogel choice can greatly impact vascular function, as ECs exhibit known sensitivity to substrate stiffness, topography, curvature, and tensile stress, and integrate these cues to regulate junctional integrity, cytoskeletal organization, and inflammatory signaling (Dessalles et al., 2021; Gordon et al., 2020; Ri et al., 2022). Matrix stiffness and composition in these systems have also been shown to directly regulate leukocyte adhesion, extravasation efficiency, and inflammatory polarization, with stiffer matrices promoting increased adhesion and migration (Ri et al., 2022; Pérez-Rodríguez et al., 2021). Hydrogel granularity has also recently emerged as an influential parameter in microvascular modeling. For example, in a recent study led by the Segura group, we observed that endothelial cells cultured in microporous annealed particle (MAP) hydrogels exhibit enhanced migration, network stability, and perfusion compared to bulk hydrogels (Anderson et al., 2025). When integrated with microfluidic MPS, such materials provide a promising platform for modeling vasculogenesis in dynamic contexts such as wound healing. These considerations are also relevant to lymphatic MPS, as tuning stiffness, matrix density, matrix composition, and granular hydrogel porosity can all impact lymphangiogenesis and lymphatic function (Alderfer et al., 2024; Henderson et al., 2021; Lugo-Cintron et al., 2020; Montes et al., 2025).

Most microvascular MPS to date have relied mainly on fibrin, with some incorporating collagen or collagen-fibrin composites, since these gel via simple thermal or enzymatic crosslinking and present native adhesion motifs that support vasculogenesis without added functionalization (Copes et al., 2019; Wang et al., 2021). However, in these materials, stiffness, degradability, and ligand density are all coupled to polymer concentration, making it difficult to tune one property independently to assess its effect on vascular cell behavior (Wang et al., 2021; Brown et al., 2020). Synthetic hydrogels—based on PEG or dextran and conjugated with RGD or collagen/fibrin-derived peptides—begin to address this limitation by allowing independent control over physical and biochemical parameters, but have historically supported less robust vascular network formation and carry their own drawbacks, including photoinitiated or multi-step crosslinking chemistries and swelling behavior incompatible with confined MPS geometries (Brown et al., 2020; López-Gutierrez et al., 2023). Progress towards incorporating MMP-cleavable linkages and designing synthetic gels with neutral swelling properties will help overcome these barriers for modeling microvasculature in MPS (Brown et al., 2020; Friend et al., 2023; Pruett et al., 2026). Ultimately, the choice between natural, synthetic, and hybrid matrices should be guided by the biological question at hand: natural matrices remain advantageous when native ECM composition or remodeling is itself under study (e.g., fibrosis or ECM-degradation models), while synthetic and hybrid matrices better suit applications requiring independent control over mechanical or biochemical parameters, including the dynamic, time-dependent behaviors discussed below.

In macrovascular MPS, where structural integrity and mechanical loading are critical considerations, materials must withstand elevated burst pressures and cyclic strain while preserving physiologically relevant compliance (Chang et al., 2026; Konig et al., 2009). Many arterial fabrication methods stem from efforts focused on engineering transplantable vascular grafts and have since been repurposed for MPS design (Luo et al., 2024). Most common designs include electrospun silk or fibrin scaffolds due to their fibrous architecture and relative anisotropic mechanics, while synthetic elastomers and reinforced hydrogels are often required to achieve arterial-range pressures (Table 1) (Fleischer et al., 2020). Notably, recent small artery MPS designs utilizing tubular scaffolds constructed from gelatin microribbons were able to achieve circumferential alignment of smooth muscle cells in the adventitia, a phenotype previously difficult to recapitulate *in vitro* (Mastoor et al., 2024). It is additionally important to note, native blood vessels exhibit non-linear elasticity, characterized by strain-stiffening behavior driven by elastin engagement at low strain and collagen recruitment at higher strain (Figure 3A) (AMRPN, 2013). However, failure to recapitulate this non-linearity can distort the transmission of wall strain and shear stress sensed by ECs and SMCs (Dalbosco et al., 2024; Wang et al., 2024). Though capturing this mechanical behavior has been a central focus of many tissue-engineered scaffolds for engraftment, only few MPS have successfully achieved this non-linear elasticity, and it is thus a necessary next step for advancing many arterial MPS models. Towards achieving this goal, computational models for evaluating arterial micromechanics may be able to inform MPS geometry, wall thickness, and material selection (Dalbosco et al., 2024).

**FIGURE 3:**
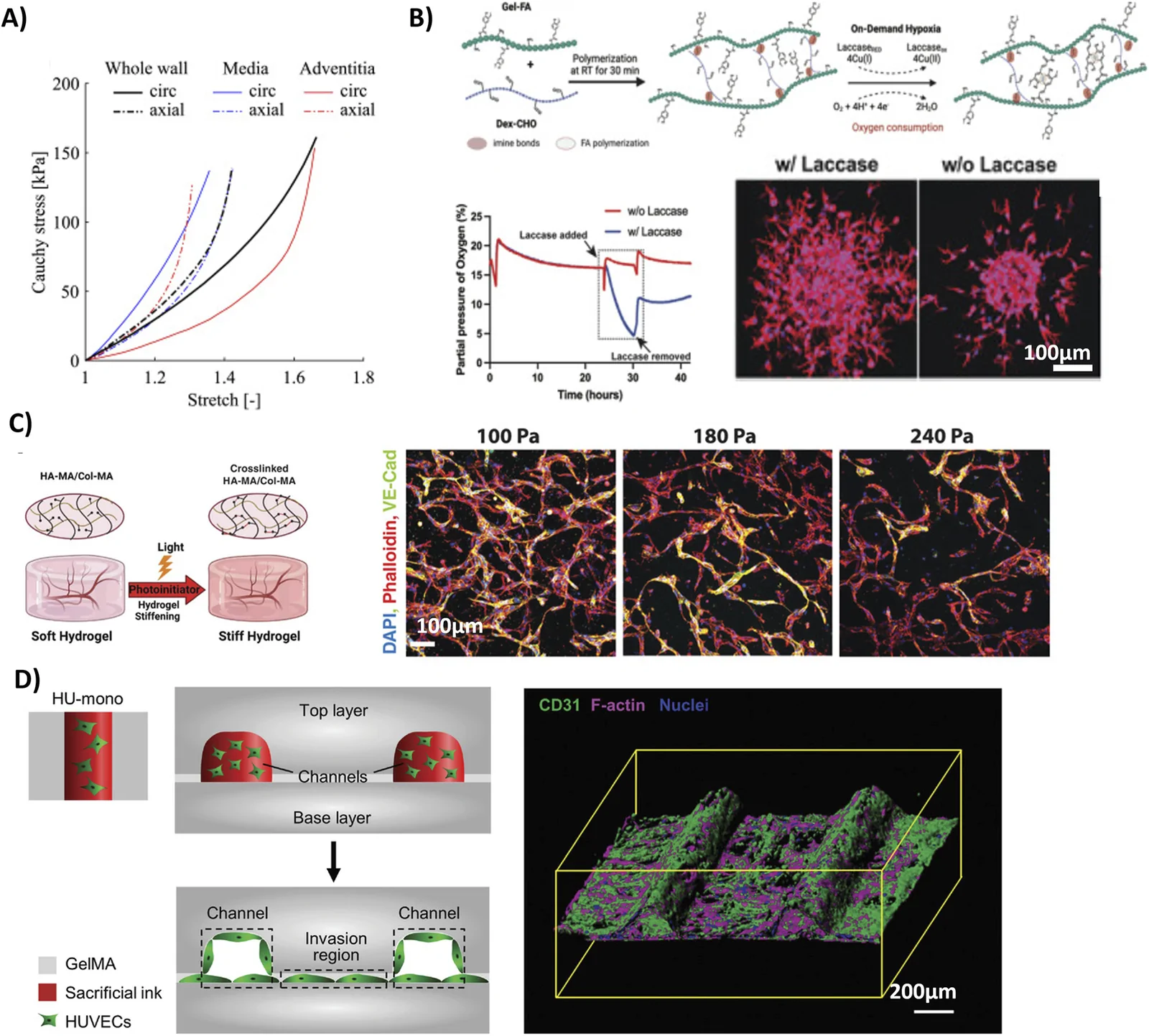
Considerations for and advancements in biomaterials to be leveraged in vascular MPS design. **(A)** Uniaxial stress response of native thoracic aorta demonstrating non-linear elasticity of the vessel, particularly the adventitia. **(A)** is reproduced from Dalbosco et al. (Dalbosco et al., 2024) under http://creativecommons.org/licenses/by/4.0/. **(B)** On-demand hypoxia inducible gelMa-based hydrogel reaches PO2 < 5% after administration of laccase and demonstrates increased endothelial (red) sprouting in an angiogenic manner. Scale bar is 100 microns. **(B)** is reproduced from Ntekoumes et al. (Ntekoumes et al., 2025) under http://creativecommons.org/licenses/by/4.0/. **(C)** Photosensitive HA-MA-Col-MA hydrogel undergoes dynamic stiffening in response to UV light and showcases subsequent decreased contractility and endothelial network regression. Scale bar is 100 microns. **(C)** is reproduced from Schnellmann et al. (Schnellmann et al., 2022) under http://creativecommons.org/licenses/by/4.0/. **(D)** Schematic illustration and fluorescent image of novel 3D printed vascular construct with gelMa, sacrificial HA, and HUVECs. HUVECS assemble into distinct vascular compartments otherwise difficult to achieve using canonical gel casting methods. Scale bar is 200 microns. **(D)** was reproduced from Orellano et al. (Orellano et al., 2022) under http://creativecommons.org/licenses/by/4.0/.

In addition to static material properties, dynamic and adaptive biomaterials introduce additional layers of complexity and physiological relevance to vascular MPS, particularly for modeling disease progression and remodeling. For example, it is known that both micro and macro-vascular microenvironments undergo substantial stiffening during several pathological processes such as aging, fibrosis, solid tumor growth, vascular calcification, and atherosclerosis (Zhang L. et al., 2025; Schnellmann et al., 2022). Employing materials capable of time-dependent stiffening in a vascular MPS can contribute to our understanding of how these changes in matrix mechanics drive pathological phenotypes or dysfunction. Our group has developed a microvascular model using a photosensitive hydrogel with methacrylated HA (HA-MA) and methacrylated collagen (Col-MA) hydrogel capable of on-demand dynamic stiffening. We have observed that dynamic stiffening leads to increased EC contractility and destabilized adherens junctions (Figure 3C) (Schnellmann et al., 2022) and promotes EC network senescence in a mechanism that is independent of canonical inflammatory pathways, decoupling mechanical inputs from biochemical ones (Song et al., 2025). Beyond time-dependent stiffening, the native vascular extracellular matrix (ECM) exhibits additional dynamic mechanics through both plastic and viscoelastic behaviors. Importantly, ECM plasticity varies substantially across tissues and is frequently reduced during disease progression, such as fibrotic remodeling, where excessive crosslinking limits matrix deformation (Jia et al., 2021). Recent biomaterial strategies decouple plasticity from elastic modulus, enabling targeted investigation of how ECM dynamics regulate vascular formation and homeostasis. In collaboration with the Xu group, we demonstrated that collagen-hyaluronic acid hydrogels functionalized with 1-adamantaneacetic acid and cyclodextrin to introduce tunable guest-host interactions regulate endothelial sprouting and vascular network organization independent of stiffness (Wei et al., 2023). In parallel, viscoelasticity is an increasingly recognized regulator of endothelial sprouting, vascular network remodeling, and mechanotransduction (Qian and Yao, 2026; Chaudhuri et al., 2015; Mierke, 2022). The native ECM is inherently viscoelastic, exhibiting stress relaxation in response to cell-generated forces, and this property varies with age and disease. Recent studies by our group and others have focused on isolating how viscoelasticity affects EC behavior, separate from modulus effects by incorporating dynamic covalent bonds in gelatin or elastin-like PEG systems to generate materials with variable rates of stress relaxation. These studies show that increased viscoelasticity promote integrin clustering, formation of focal adhesion kinases, MMP activation and ultimately, robust vasculogenesis through YAP/TAZ signaling pathways (Wei et al., 2020; Keshavarz and Smith, 2024; Shayan et al., 2023). Although these platforms leveraged dynamic covalent bonds to achieve viscoelastic behavior, additional methods include modulating physical cross-linking via guest-host, hydrophobic, or ionic interactions, and incorporating these strategies into vascular MPS may yield further insights into microvascular network formation and stability (Qian and Yao, 2026).

Dynamic materials are not limited to mechanics alone, however. For example, our lab has developed a tunable, on demand, hypoxia-inducible hydrogel to investigate the effects of hypoxia (Blatchley et al., 2021) (oxygen deprivation) on network formation (Park and Gerecht, 2014; Blatchley et al., 2019). Building on these foundations, we recently established an on-demand hypoxic hydrogel to demonstrate the effect of acute rapid hypoxia on endothelial sprouting (Ntekoumes et al., 2025) (Figure 3B). This material offers temporal control over oxygen tension, a key feature of ischemic tissues in CVD, and has been shown to regulate angiogenesis, barrier maturation, and inflammatory activation in microvascular beds such as the retina and tumor vasculature (Blatchley et al., 2021). Collectively, these dynamic material strategies are particularly well suited to capturing the adaptive behavior of micro- and mesovascular tissues, while macrovascular applications remain constrained by the need to balance adaptability with structural robustness.

Lastly, advances in 3D printing and biofabrication have further expanded the design space for vascular MPS by enabling precise control over geometry, material placement, and hierarchical organization across vascular scales. Though 3D printing itself is not new to the field of vascular MPS development, recent advances in printable hydrogel chemistries–including yield-stress support baths, ECM-derived inks, and stimuli-responsive materials–emphasize a shift from static channel fabrication toward materials enabling vascular remodeling (Kim and Cho, 2024; Son et al., 2025). Zhang et al. introduced clickable PEG-norbornene (PEGNB) microbeads as a suspension-printing support bath that can be secondarily crosslinked into a mechanically stable MAP scaffold. This enabled sacrificial bioprinting of mesoscale channels (∼50–1,000 µm) for pre-pattered endothelial-pericyte networks in a granular matrix (Zhang IW. et al., 2025). Additionally, Orellano et al. recently leveraged multi-material projection-based stereolithography to spatially pattern GelMA and a degradable HA-MA sacrificial ink, creating compartments with distinct mechanics and degradability to direct endothelial self-organization (Figure 3D) (Orellano et al., 2022). Importantly, this showcased the use of soft, stress-relaxing materials to form endothelial networks of defined geometries without the need for pre-patterned templates. Altogether, combined strategies leveraging informed tissue mechanics, the use of dynamic materials to model time-dependent processes, and 3D printing to achieve increasingly complex architectures have the potential to significantly advance the complexity of existing MPS and the range of possible diseases they can model.

### Hemodynamic flow

In addition to physical interactions with the surrounding matrix, vascular cells experience substantial mechanical stimuli as a result of hemodynamic forces. These forces vary across tissue types and throughout the vascular hierarchy, producing differences in hydrostatic pressure, circumferential strain, endothelial shear stress, and transmural flow through the vessel wall; recapitulating these forces is a critical component of vascular MPS. Among them, shear stress is particularly relevant to MPS design because it is determined by vessel geometry and flow conditions, allowing direct comparison between MPS and human physiology (Nahon et al., 2024). Importantly, hemodynamic shear stress is a primary regulator of vascular cell phenotype and function, and its magnitude, directionality, and temporal profile vary substantially across the vascular hierarchy (Martier et al., 2024). Arteries experience a wide range of shear stresses (∼10–70 dynes/cm^2^) with high pulsatility and oscillatory components driven by the cardiac cycle, and disturbed flow regions arising at bends and bifurcations. As flow progresses to the arterioles, pulsatility is dampened, and wall shear stress (WSS) values converge around 50 dynes/cm^2^. This eventually converges to near-laminar flow as blood reaches the capillaries, albeit with highly heterogenous WSS depending on tissue type. WSS drops dramatically as fluid flow reaches the venules (∼15 dynes/cm^2^) and veins (1–6 dynes/cm^2^) (Martier et al., 2024; Papaioannou et al., 2006). Across vascular cell types, these scale-dependent flow regimes contribute significantly to cell phenotype. ECs sense mechanical stimuli through a diverse array of components–including the glycocalyx, G-protein–coupled receptors (GPCRs), NOTCH1, P2X4, and the mechanosensitive ion channel PIEZO1 (Tamargo et al., 2023; Zeng, 2017) – and *in vitro* studies demonstrate that shear stresses around and above 10 dynes/cm^2^ are required to induce EC alignment and elongation, angiogenic sprouting and remodeling (Galie et al., 2014; Ostrowski et al., 2014), cytoskeletal reorganization, altered responsiveness to inflammatory stimuli such as tumor necrosis factor α (TNFα), and enhanced barrier integrity (Rickman et al., 2023; Linares et al., 2025). Linares et al. demonstrated that low shear stress around 1.5 dynes/cm^*2*^ can increase alignment, but the highest alignment is observed at 10 dynes/cm^*2*^ (Figure 4A) (Linares et al., 2025). Single-cell RNA sequencing further reveals that endothelial heterogeneity increases under distinct laminar, shear, and turbulent flow profiles, giving rise to EC subpopulations with discrete transcriptomic signatures (Ziqing Liu et al., 2021; Salazar-Martín et al., 2023).

**FIGURE 4:**
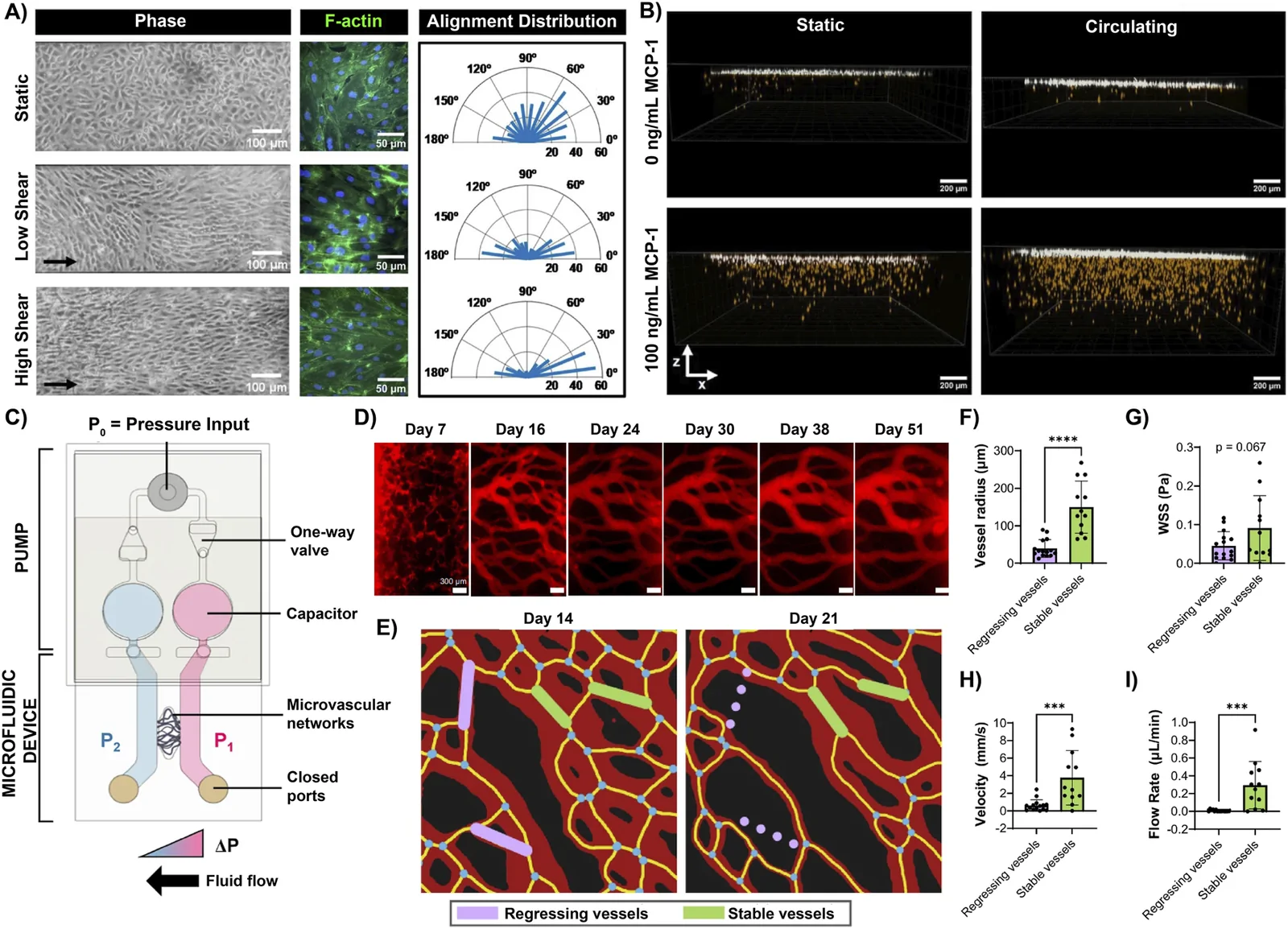
Considerations for fluid flow in vascular microphysiological systems. **(A)** Magnitude of shear stress impacts endothelial cell alignment, with 1.5 dynes/cm^2^ used for low shear stress and 10 dynes/cm^2^ for high shear stress**. (B)** Monocytes that are adhered (white) or have transmigrated through (orange) endothelial cell barriers show different behaviors with fluid flow and chemoattractants. Panels A and B were reproduced from Linares et al. (Linares et al., 2025) under the Creative Commons license, found here: http://creativecommons.org/licenses/by-nc-nd/4.0/. **(C)** Incorporation of pump and microfluidic device allows long-term culture, with **(D)** stable microvascular networks for up to 51 days **(E)** Imaging over time allows regressing and stable vessels to be identified. Vessel radius **(F)** wall shear stress **(G)** flow velocity **(H)** and flow rate **(I)** are reported for regressing and stable vessels. Panels C–I were reproduced from Floryan et al. (Floryan et al., 2025) under the Creative Commons license, found here: http://creativecommons.org/licenses/by-nc-nd/4.0/.

Hemodynamic flow, through both circumferential strain and interstitial shear as a result of transmural flow, similarly regulates SMC behavior. Indirectly, shear-induced EC production of endothelial nitric oxide synthase (eNOS) promotes nitric oxide (NO) signaling to SMCs, driving relaxation and vasodilation, whereas large fluctuations in hydrostatic pressure and stretch–such as those associated with pulsatile flow–depolarize SMCs and trigger contraction to maintain vascular tone (Xiao et al., 2023). In culture, both shear and circumferential stretch have been shown to directly influence SMC phenotype, with hemodynamic flow promoting SMC maturation, contractility and quiescence (Scott et al., 2013; Ueba et al., 1997). Likewise, SMCs exposed to aberrant flow patterns (i.e., dampened pulsatility or elevated interstitial shear) experience synthetic SMC proliferation and intimal thickening (Ueba et al., 1997; Papadaki et al., 1998; Gao et al., 2024). Beyond mural cells, flow also governs immune-vascular interactions, with EC-monocyte co-culture systems demonstrating shear-dependent regulation of monocyte adhesion, rolling, and transmigration (Linares et al., 2025). At low shear stress levels of 1.5 dynes/cm^*2*^, Linares et al. found that flow enhances chemoattractant-mediated transmigration of monocytes (Figure 4B) (Linares et al., 2025).

These studies highlight fluid flow, particularly physiologically relevant shear stresses, as crucial for maintaining proper vascular phenotypes and function, necessitating the incorporation of physiologically relevant flow profiles in vascular MPS design; this is especially significant in the context of disease modeling, a primary application of most MPS platforms. Using a microvascular MPS, Hatch and colleagues demonstrated that flow upregulates EC expression of angiotensin converting enzyme 2 (ACE2)– a receptor for the SARS-Cov-2 spike protein–resulting in substantially increased viral infiltration compared to static culture (Hatch et al., 2024). Similarly, Salazar-Martín and colleagues used single-cell RNA sequencing to show that flow profoundly alters EC responsiveness to antiproliferative drugs commonly used in drug-eluting stents, revealing flow-dependent drug sensitivity that is not captured under static conditions (Salazar-Martín et al., 2023). In the context of tumor vascularization, Hachey and colleagues employed a microfluidic platform to generate vascularized colorectal tumor organoids. The study showed that their perfused, endothelialized constructs had lower drug sensitivity than static 2D cultures, better recapitulating *in vivo* therapeutic responses (Hachey et al., 2021). In each of these instances, fluid flow was a necessary component for reproducing disease phenotypes and elucidating mechanisms governing therapeutic efficacy.

To meet these biological requirements, vascular MPS employ a range of strategies to generate physiologically relevant flow across vascular scales. In microvascular models, passive flow approaches are most commonly used due to their simplicity, laminar flow profiles, and ability to generate low shear stresses (Jin et al., 2024). Gravity-driven hydrostatic pressure gradients are frequently applied to produce unidirectional flow sufficient to support endothelial alignment and barrier function in open-channel systems (Zheng et al., 2012; Yang et al., 2019; Ono et al., 2025) or lumenization, perfusion, and long-term network stability in hydrogel-embedded microvascular models (Maurissen et al., 2024; Kim et al., 2021). Importantly, shear stress can be tuned by adjusting reservoir height, providing a straightforward method for modulating endothelial mechanostimulation (Jin et al., 2024). Rocker-based platforms extend this approach by cyclically altering fluid height to maintain perfusion, circumventing the need to replenish media reservoirs to renew the hydrostatic gradient, but doing so at the cost of introducing oscillatory or bidirectional flow (Ch et al., 2021). Recent designs from the Broek laboratory address this limitation through incorporation of an acellular bypass channel that returns fluid to the inlet of the primary culture channel, enabling net unidirectional flow across microvascular beds when operated on a rocker, and promoting EC alignment (Ehlers et al., 2025). Additional passive strategies, including surface tension-driven and osmosis-driven flow, offer pump-free alternatives for maintaining perfusion in enclosed microfluidic systems, though these methods have primarily been used to model perfusion in tissues such as the brain, pancreas, and connective tissues, rather than the microvasculature and their use has declined with the availability of more robust alternatives (Jin et al., 2024; Park et al., 2009; Xing et al., 2016; Choi et al., 2013; Jun et al., 2019). Owing to the limited mixing inherent to laminar flow, passive flow architectures with interconnected channels can also be used to generate stable chemical gradients for modeling endocrine or cytokine signaling and spatially patterned drug delivery (Samandari et al., 2021; Liu Y. et al., 2023). Nevertheless, passive systems offer limited control over pulsatility, flow stability, and long-term scalability, restricting their utility for macrovascular modeling and disease states associated with disturbed flow.

Active pumping strategies have therefore been widely adopted to introduce greater hemodynamic complexity and long-term perfusion control. Syringe pumps are commonly used to impose well-defined, unidirectional laminar flow across endothelialized channels, particularly in arteriole and venule/venous MPS (Akbari et al., 2019; Mirzapour-Shafiyi et al., 2025), but are limited by finite reservoir volumes, making extended culture or continuous drug dosing challenging. Peristaltic pumps enable closed-loop circulation, supporting long-term perfusion and repeated drug administration while more closely approximating pulsatile arterial flow. These systems are particularly well suited for macrovascular MPS, where cyclic strain and interstitial flow are dominant cues (Gao et al., 2024; Helms et al., 2021; K et al., 2009); however, their reliance on external tubing–known to adsorb proteins and small-molecule drugs–and large media volumes can be limiting. To address these challenges, several groups, including the Toh and Yilmazer laboratories, have developed miniaturized peristaltic pumps specifically optimized for MPS and organ-on-chip applications (Cataño et al., 2023; Panatli et al., 2025). These systems incorporate custom printed circuit boards (PCBs) that allow user-programmable flow profiles while minimizing dead volume and improving compatibility with standard incubator workflows. A particularly notable advancement is the MicroHeart developed by the Kamm laboratory, which uses a pneumatically actuated fluid capacitor to generate recirculating flow through a compact, integrated platform (Offeddu et al., 2021). Using this system (Figure 4C), the Kamm group achieved sustained perfusion and culture of self-assembled microvascular networks for up to 51 days (Figure 4D) – far exceeding the typical 14-day stability of most vascular MPS–and demonstrated that long-term flow rescues network regression, increases flow velocity, promotes morphological homeostasis and endothelial quiescence, and reduces inflammatory signaling (Floryan et al., 2025). Floryan et al. also demonstrated that this system was capable of tracking vessel stability over time (Figure 4E), finding differences in vessel radius (Figure 4F), wall shear stress (Figure 4G), velocity (Figure 4H), and flow rate (Figure 4I) between stable and regressing vessels (Floryan et al., 2025). These findings highlight that not only can flow impact vascular function, but quantifying flow in vasculature of varying stability could enhance our understanding of microvascular remodeling. Cook et al. also recently developed an open-source (Cook et al., 2025), tubing-free 3D printed MPS system with the ability to connect two tissues or hydrogel constructs with recirculating flow driven by an impeller pump (Cook S et al., 2025) that could be applied for future study of crosstalk between vascular systems and other tissues.

Beyond uniform flow, increasing attention has been directed toward modeling disrupted and spatially heterogeneous flow. Disrupted flow is a hallmark of numerous vascular diseases such as atherosclerosis, pulmonary arterial hypertension, many forms of liver and kidney disease, venous thrombosis, and vascular aging (Martier et al., 2024). In response to disrupted flow, oscillatory shear stress, and reduced laminar shear stress, ECs shed components of the glycocalyx, reducing mechanotransduction of shear and increasing leukocyte adhesion, undergo endothelial-to-mesenchymal transition (endMT) or adopt otherwise pro-inflammatory, pathologic phenotypes, and exhibit decreased barrier integrity (Dessalles et al., 2021; Zeng, 2017; Estrada et al., 2011; Baratchi et al., 2017; Andueza et al., 2020; Shinohara et al., 2025). Bypass and stenosis-mimicking platforms aim to capture these phenomena through incorporation of bifurcating channels, circular blocks, and intersecting media inlets to generate regions of flow separation, recirculation, and oscillatory shear characteristic of disrupted arteries, arterioles, and venules (Akbari et al., 2019; Balaguru et al., 2016; Xiao et al., 2024). Importantly, deep veins exhibit flow profiles distinct from those of other vessels in the vascular hierarchy due to the presence of bicuspid flaps that act as valves, though few vascular MPS incorporate this feature. In instances of reduced flow, blood can accumulate behind these valves, forming hemostatic pockets that have been shown to contribute to the pathogenesis of deep vein thrombosis (Suresh et al., 2026). Modern vein-on-chip platforms, such as those developed by the Brill and Jain groups, incorporate endothelial cells (ECs) within microfluidic devices featuring venous valvular geometries, enabling mechanistic investigation of thrombus initiation (Rajeeva Pandian et al., 2020; Baksamawi et al., 2023). Complementing experimental advances, computational modeling has become an integral component of modern vascular MPS design, enabling quantitative prediction and optimization of flow fields across complex geometries. Computational fluid dynamics (CFD) in combination with optical coherence tomography (OCT) can be applied to resolve velocity and perfusion heterogeneity, shear gradients, and network-level resistance, metrics that are difficult to obtain experimentally in models with disrupted flow profiles (Mirzapour-Shafiyi et al., 2025; Sung et al., 2019; Zhao et al., 2021; Ham et al., 2023; Gruionu et al., 2024; Rajeeva Pandian et al., 2026; Rota et al., 2023). Such models are increasingly used to validate and guide vascular MPS design, optimize inlet-outlet configurations, and tune perfusion parameters to achieve physiologically relevant shear distributions. Notably, the Cattaneo group used a 3D model of a patient-specific carotid artery bifurcation to design a thin-walled hydrogel artery for precision analysis of fluid velocity, shear, and arterial distension, demonstrating how patient-specific geometries can inform model design (Shiravand et al., 2024). Similarly, the Ju group recently used an MRV scan of a patient’s cerebral vasculature to generate a personalized Vein-Chip model of a venous stenosis for investigating Virchow’s triad and its contributions to cerebral venous sinus thrombosis in a patient specific manner (Zhao et al., 2023).

Finally, while the flow strategies above are largely validated using standard culture medium, an emerging and biologically distinct challenge is the incorporation of whole blood or its formed components (i.e., platelets, erythrocytes, coagulation factors, red blood cells) into vascular MPS. Unlike cell culture media, blood is a non-Newtonian fluid with distinct rheological properties that necessitate consideration in certain vascular disease models. Namely, the apparent viscosity of blood is highly dependent on the density of red blood cells or hematocrit and on vessel diameter, particularly as diameters decrease below 300μm, generating shears that deviate from the geometry-derived values with cell culture media alone (Salame and Fenech, 2025; Johnston et al., 2004; Sec et al., 2013). Introducing blood also raises device-level hemocompatibility concerns absent from medium-only systems: common substrate materials (e.g., PDMS, polycarbonate) can drive fibrinogen adsorption, platelet activation, and hemolysis at the blood-material interface, motivating biomimetic endothelial linings or antifouling polymer or heparin-based surface treatments to reduce artifactual clotting (Goh et al., 2024; Newman et al., 2023). Common surface treatments derive largely from approaches used for blood-contacting biomedical devices such as zwitterionic polymers, albumin, PEG, or heparin coatings shown to prevent non-specific protein adsorption (Gholizadeh and Benam, 2026). Heparin coatings are particularly effective due to their ability to inhibit coagulation cascade factors (Biran and Pond, 2017), though they raise concerns in the disease modeling context as their anticoagulation behavior may mask important biological phenomena. Despite these challenges, blood-perfused vessel-on-chip platforms have proven valuable for disease modeling in contexts where systemic blood components are mechanistically central, including thromboinflammation, drug-induced thrombocytopenia, COVID-19-associated microthrombosis, and sickle cell disease, where whole-blood perfusion under physiological flow has revealed altered flow dynamics and vaso-occlusive behavior not captured by acellular or medium-based models (Harada et al., 2024; Weener et al., 2024; Manz et al., 2020). As MPS platforms increasingly target these blood-dependent pathologies, standardizing approaches to hemocompatibility, rheology, and anticoagulation will be an important direction for the field.

## Conclusion and future directions

Overall, exciting progress in the field of vascular MPS marks a transition toward ever-increasing model complexity, expanding our ability to accurately recapitulate vascular function and dysfunction. Improved models are leading the way toward enhanced discovery and screening of clinically relevant therapeutics. Multicellular models better match the cellular complexity seen in tissues, while tunable ECMs and dynamic fluid flow allow for precise replication of relevant physical cues. It is important to note that while there are numerous methods for increasing the relevance and complexity of vascular MPS, priority should be given according to the intended application or disease being modeled. Microvascular barrier models such as the blood-brain or blood-retinal barriers may place greater emphasis on cell selection to achieve proper junctional integrity and accurate permeability coefficients, whereas thrombosis and atherosclerosis models may prioritize strain-tolerant biomaterial selection, accurate vessel geometries, and blood-like hemodynamics to mimic pathological shear and coagulation over long-term culture. Selecting the appropriate level of complexity therefore requires matching each added feature–additional cell types, tunable ECM, disease-relevant flow regimes, or blood components–to specific mechanistic or functional readouts, and validating that the resulting model reproduces the relevant physiological or pathological benchmark (e.g., a target permeability coefficient, shear stress range, or clotting kinetics).

In addition to the significant improvements in individual vascular MPSs, a critical next step for the field is integrating multiple levels of the vascular hierarchy into unified platforms. Many vascular diseases do not arise or act at a single spatial scale; pathologies such as peripheral artery disease, thrombosis, and hypertension originate in, or primarily affect larger vessels, but ultimately manifest as impaired perfusion, inflammation, and tissue injury at the microvascular level (Margolis et al., 2023). Therapeutic interventions targeting one vascular compartment–such as stents, vasodilators, or anti-thrombotic agents–inevitably alter hemodynamics and cellular responses throughout the downstream vascular tree and warrant sufficient *in vitro* characterization. Additionally, a central goal of *in vitro* vascularization strategies is to support the survival and function of engineered tissues and organoids, which, once they exceed diffusion limits (∼200 μm), require not only capillary networks but also upstream arteriolar and arterial conduits to ensure sufficient perfusion (Jain et al., 2005).

While microvascular MPS are widely being developed and large-diameter (>6 mm) tissue-engineered blood vessels (TEBVs) consistently demonstrate long-term patency and mechanical robustness, small artery and arteriole/venule-scale models have historically been underrepresented and will be a necessary component of advancing towards hierarchical vascular integration (Fleischer et al., 2020). Recent studies are beginning to address this gap across a range of mesovascular length scales. At diameters on the order of ∼10 μm, some platforms adapt established microvascular systems and apply elevated cyclic shear to induce self-assembled arteriole-like structures (Shivani et al., 2025). At intermediate scales (∼100 μm), current approaches generally diverge between endothelialization of hollow hydrogel channels within microfluidic devices (Maringanti et al., 2024; Piombo et al., 2025) and the seeding of endothelial and mural cells onto self-supporting fibrin and collagen tubular constructs cultured in perfusable bioreactors (Abutaleb et al., 2025; Yarbrough et al., 2026). For vessels approaching 1–6 mm in diameter, many platforms continue to rely on synthetic materials to achieve the mechanical robustness required for sustained perfusion, although select systems have demonstrated successful *in vivo* transplantation (Zhang J. et al., 2025), highlighting that full biomimicry of all structural arterial components may not be a necessary precursor for functional vascular modeling nor their integration in future hierarchical MPS.

Toward full hierarchical integration, many groups have developed arteriole-capillary interfaces through endothelial sprouting to and from hollow tubes or channels with endothelialized lumens (Margolis et al., 2021; Debbi et al., 2022; Song et al., 2020; Szklanny et al., 2021). One notable advancement on this approach comes from the Levenberg group, which achieved artery-microvascular integration by a using a combination of synthetic and 3D printed biomaterials. They fabricated a large porous Poly-L-lysine (PLL)/Poly(lactic-co-glycolic acid) (PLGA) artery scaffold surrounded by patterned 3D printed human collagen methacrylate channels seeded with HUVECs to promote microvascular network formation (Szklanny et al., 2021). Using this approach, they have been able to generate vascularized muscle and fat tissue-engineered flaps for *in vivo* applications (Fischer et al., 2026). In 2023, the Kamm group utilized this method to establish a tri-compartment vascular MPS encompassing arteriole, capillary, and venule-specific vessels and architectures (Chen S et al., 2023). Another common approach towards hierarchical integration is the repatterning of macro and micro vascular channels. For example, the Chen group is utilizing sacrificial scaffolds constructed from gallium to construct vascular trees in collagen gels ranging from 300 μm in diameter to 30 μm (Sundaram et al., 2024). Excitingly, the Böer group recently developed a three-scale, fully perfusable vascular model by combining mechanically robust fibrin-based large vessels with softer, cell-laden fibrin hydrogels for endothelial network formation. Their MPS utilizes orthogonally patterned macro- and meso-scale lumens to enable hierarchical flow down to a capillary level and serves as a compelling example of successful multi-vascular-scale integration (Helms et al., 2022).

In parallel, there is growing momentum toward the development of organ- and tissue-specific vascular MPS that explicitly account for the unique structural, mechanical, and barrier properties of specialized vascular beds (Nam et al., 2024). These platforms leverage EC co-culture with organ-relevant parenchymal or stromal cell types and increasingly incorporate tissue-specific endothelial phenotypes, physiologically appropriate flow regimes, and tailored ECM environments to more accurately recapitulate organ function and disease susceptibility. Recent organ-specific vascular MPS have been used to investigate aberrant EC-podocyte interactions in glomerular capillaries during kidney disease (Kroll et al., 2024; Bhattacharya et al., 2025); elucidate how EC co-culture and shear stress regulate cardiomyocyte contractility in *in vitro* cardiac tissue models (Liu et al., 2024); model endothelial-to-mesenchymal transition (endMT) of liver sinusoidal endothelial cells and identify mechanisms contributing to liver fibrosis and metabolic liver disease (Whiteford et al., 2022); investigate the role of endothelial-epithelial cross talk and dysfunction in lung and gastrointestinal diseases (Lia et al., 2024; Kim et al., 2026; Sharma et al., 2024), assess the permeability of, and identify vascular shuttling proteins mediating transport across, the blood-brain barrier (Hajal et al., 2021; Choi et al., 2024); and even model diseases that selectively target the vasculature of the inner blood retinal barrier (Maurissen et al., 2024). Notably, recent advances in modeling the inner blood retinal barrier, such as the development of retinal-specific EC differentiation protocols (Lin et al., 2026), in combination with existing models for the outer blood retinal barrier (Song et al., 2022) underscore the ability of vascular MPS to resolve compartment-specific transport and inflammatory mechanisms in retinal diseases like diabetic retinopathy or macular degeneration that are largely inaccessible *in vivo*. As these organ-specific vascular systems continue to mature, a key next step will be integrating them with existing parenchymal models that capture other essential components of organ function but currently lack vasculature. Such integration will be critical for building toward true organ-on-chip platforms and interrogating reciprocal vascular-tissue signaling and whole-organ-level pathophysiology.

Complementary to vascular hierarchy and tissue-specific vascular MPS, models of the lymphatic vasculature have also increased in number and complexity and have even been combined with blood vasculature (Cao et al., 2019; Knezevic et al., 2017). The lymphatic system experiences unique physical forces, as interstitial fluid flow drives lymph collection, exposing lymphatic endothelial cells to both shear stresses and bulk flow forces. Interstitial fluid flow is a key consideration in these systems, as it can alter immune cell migration, inflammatory cytokine secretion, lymphangiogenesis, and barrier function (Hammel et al., 2025; Ilan et al., 2023). Modeling the lymphatic system offers a unique opportunity to apply the many considerations discussed in this review, particularly because specialized lymphatic systems are found in many distinct tissues throughout the body, such as the lymph nodes, meninges, and gut. Additionally, disorders such as lymphedema (Song et al., 2022), cancer metastasis (Stacker et al., 2014), traumatic brain injury (Bolte et al., 2020), and Alzheimer’s disease (Da et al., 2018) can all involve dysfunctional lymphatic systems. Implementing tissue-mimicking biomaterials, increasing the inclusion of tissue-specific cell types, and incorporating multiple types of fluid flow will enhance modeling of the lymphatic system in MPS.

Finally, increasing throughput and analytical depth represent key enabling directions for the translational deployment of vascular MPS. Recent demonstrations from the Vunjak-Novakovic and Luo groups of multi-organ MPS platforms, so-called body-on-a-chip platforms, incorporating vascular flow across anywhere from 4 to 18 tissues illustrate the feasibility of scaling these systems for drug screening and systemic toxicity assessment (Ronaldson-Bouchard et al., 2022; Wang et al., 2025). Concurrently, the integration of single-cell transcriptomics and spatial omics into vascular MPS workflows offers unprecedented resolution for dissecting cell-state heterogeneity, cellular responses to localized biophysical and biochemical cues, and mechanotransduction pathways. Single-cell RNA-seq resources such as the Human Vascular Atlas can inform specific biomarkers for tissue- and organ-specific specification of vascular cell types in vascular MPS; the use of computational inference pipelines like scFate or Drug2Cell can help to elucidate potential transition states between cell types during disease progression and the likelihood of drug-receptor interactions in drug screening platforms (Barnett et al., 2024); and methods like MAGIC-seq–grid-pattern, microfluidic bar-coding–may enable spatio-transcriptomic mapping across complex (multicellular or diverse architecture) MPS (Du and Yang, 2025). When combined with isogenic cell sources and controlled microenvironments, these approaches will enable data-driven model validation and refinement. Together, continued advances in hierarchical integration, tissue specificity, throughput, and multi-omic analysis are expected to further establish vascular MPS as indispensable platforms for precision vascular biology and next-generation therapeutic development.

## Glossary

ACE2: Angiotensin converting enzyme 2
CCC: Charcoal stripped serum
CFD: Computational fluid dynamics
Col-MA: Methacrylated collagen
CVD: Cardiovascular disease
EC: Endothelial cell
ECM: Extracellular matrix
endMT: Endothelial-to-mesenchymal transition
eNOS: Endothelial nitric oxide synthase
FBS: Fetal bovine serum
GelMA: Gelatin methacryloyl
GPCRs: G-protein–coupled receptors
HA-MA: Methacrylated hyaluronic acid
HUVECs: Human umbilical vein endothelial cells
iECs: Induced pluripotent stem cell derived endothelial cells
iPSC: Induced pluripotent stem cells
iSMCs: Induced pluripotent stem cell derived smooth muscle cells
LEC: Lymphatic endothelial cell
MAP: Microporous annealed particle
MPS: Microphysiological systems
NO: Nitric oxide
OCT: Optical coherence tomography
OoC: Organ-on-chip
PCB: Printed circuit boards
PEG: Poly(ethylene glycol)
PEGNB: PEG-norbornene
PLGA: Poly(lactic-co-glycolic acid)
PLL: Poly-L-Lysine
SMC: Smooth muscle cell
TEBV: Tissue-engineered blood vessel
TGF-β: Transforming growth factor-β
TNFα: Tumor necrosis factor α
αSMA: α-smooth muscle actin
